# Moisture harvesting and water transport through specialized micro-structures on the integument of lizards

**DOI:** 10.3762/bjnano.2.24

**Published:** 2011-04-13

**Authors:** Philipp Comanns, Christian Effertz, Florian Hischen, Konrad Staudt, Wolfgang Böhme, Werner Baumgartner

**Affiliations:** 1 Department of Cellular Neurobionics, RWTH-Aachen University, Lukasstr. 1, 52056 Aachen, Germany; 2 Department of Experimental Physics Ia, RWTH-Aachen University, Sommerfeldstr., 52056 Aachen, Germany; 3 Zoologisches Forschungsmuseum Alexander Koenig (ZFMK), Adenauerallee 160, 53113 Bonn, Germany

**Keywords:** capillary, horned lizard, rain harvesting, thorny devil, water transport

## Abstract

Several lizard species that live in arid areas have developed special abilities to collect water with their bodies' surfaces and to ingest the so collected moisture. This is called rain- or moisture-harvesting. The water can originate from air humidity, fog, dew, rain or even from humid soil. The integument (i.e., the skin plus skin derivatives such as scales) has developed features so that the water spreads and is soaked into a capillary system in between the reptiles' scales. Within this capillary system the water is transported to the mouth where it is ingested. We have investigated three different lizard species which have developed the ability for moisture harvesting independently, viz. the Australian thorny devil (*Moloch horridus*), the Arabian toadhead agama (*Phrynocephalus arabicus*) and the Texas horned lizard (*Phrynosoma cornutum*). All three lizards have a honeycomb like micro ornamentation on the outer surface of the scales and a complex capillary system in between the scales. By investigation of individual scales and by producing and characterising polymer replicas of the reptiles' integuments, we found that the honeycomb like structures render the surface superhydrophilic, most likely by holding a water film physically stable. Furthermore, the condensation of air humidity is improved on this surface by about 100% in comparison to unstructured surfaces. This allows the animals to collect moisture with their entire body surface. The collected water is transported into the capillary system. For *Phrynosoma cornutum* we found the interesting effect that, in contrast to the other two investigated species, the water flow in the capillary system is not uniform but directed to the mouth. Taken together we found that the micro ornamentation yields a superhydrophilic surface, and the semi-tubular capillaries allow for an efficient passive – and for *Phrynosoma* directed – transport of water.

## Introduction

Arid environments are characterized by low precipitation and can therefore only be used as habitats if the vitally necessary water is accessible to the respective organism. To this end, two major challenges must be overcome: (1) Water (in its available form) must be collected, and (2) it must be transported to the place of ingestion. It is well known that not only insects (e.g., the famous Namibian tenebrionid beetles) but also several deserticolous and savanicolous lizards are able to collect water with their integument, and this ability has been termed "rain harvesting" [1–2]. It consists of two elements: A specific behaviour combined with special body postures, and a particular (micro-) morphology of the integument allowing the collection and transport of water towards the mouth. A translation of the German term "Feuchtigkeitsernten", i.e., "moisture harvesting", appears more appropriate to us as it describes the different kinds of water acquisition more comprehensively. It is important to note that, for the reptiles concerned, no significant water uptake is done through the integument itself. Rather, the water is transported on the integumental surface towards the mouth, performed by means of particular micro structures. As most of these lizards are unable to lick water from most parts of their body, such a water transport mechanism is essential for them. The stereotypic moisture harvesting behaviour of the Texas horned lizard *Phrynosoma cornutum* (Iguanidae: Phrynosomatinae) was extensively studied and described by [1]. However, other lizards such as the Australian thorny devil (*Moloch horridus*, Agamidae) lack such behavioural traits but nonetheless perform moisture harvesting in a successful way [2–5].

As well as *Phrynosoma* and *Moloch* [6–8], moisture harvesting has further been observed in the agamid genera *Phrynocephalus* [9] and *Trapelus* [10], and also in an arid-adapted testudinid tortoise, viz. *Psammobates tentorius trimeni* [11]. As – apart from the tortoise – the lizard species mentioned above are not closely related to each other, their ability to harvest moisture from their terrestrial environment must have evolved convergently or, in other words, has been "invented" several times during their evolution.

Generally, the integument (from Latin *integere* = to cover) is the organ system that covers the body and protects it from water loss and/or damage. The integument consists of the skin and its derivatives such as scales, feathers, hairs and nails and has a variety of functions: Next to mechanical protection and prevention of water loss from lower tissue layers, it serves also for temperature regulation and as a transmitter for tactile stimuli. The integument of lizards consists of several layers [12–13]: An outer beta-keratin layer mostly composed of beta-keratin, an inner alpha-layer built up from alpha-keratin and a meso layer separating the two former layers. The beta-layer is covered by the so-called "Oberhäutchen" which often exhibits particular micro structures (microdermatoglyphics). Several lizard species capable of moisture harvesting exhibit a honeycomb-shaped micro structure [14]. Next to the honeycomb-micro ornamentation, a special property of moisture-harvesting lizards is the existence of micro-channels (or interscalar channels) [4,15] formed by the partially overlapping (imbricate) scales. These channels have a narrow opening on their superficial side and thus form a semi-tubular capillary system over the entire lizard's body. This capillary system was believed to serve for the transport of water towards the mouth where the active water ingestion takes place [4,8].

Apart from the biological relevance, the exact functional morphology of lizard integumental structures allowing for moisture harvesting might also be of technical interest wherever efficient collection of small amounts of liquids and/or passive transport of these liquids is required. To gain a deeper insight of and understanding for moisture harvesting, we investigated the micro morphology of the skin of three lizard species known to perform moisture harvesting, viz. the iguanid *Phrynosoma cornutum*, and the two agamids *Moloch horridus* and *Phrynocephalus arabicus*. We further tried to mimic the properties and effects of the natural integument by manufacturing replicas of the surface topography. We found that in fact this micro ornamentation yields a super-wettable (superhydrophilic) surface, and the semi-tubular capillaries allow for an efficient – and in the case of *Phrynonoma cornutum* even directed! – passive transport of water.

## Results and Discussion

### Macroscopic morphology and wettability of the lizards' integuments

The three species under investigation were chosen because all perform moisture harvesting but developed separately in different arid areas. *Phrynosoma cornutum* developed in the deserts and steppe of North America, *Moloch horridus* is found in the Australian deserts and *Phrynocephalus arabicus* inhabits arid areas in the Near East and the Arabian peninsula. Thus we hoped that common structures among these species serve for the common goal of water acquisition.

**Figure 1:**
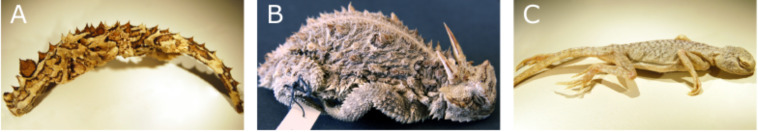
The three lizard species under investigation. (A) *Moloch horridus* with an array of spikes covering the entire upper side of the body. The snout–vent length is about 90 mm and the total length is about 140 mm. (B) *Phrynosoma cornutum* also exhibits thorny appendages. The snout–vent length is about 95 mm. (C) *Phrynocephalus arabicus* has a velvet like surface and no apparent protuberations. The snout–vent length is about 80 mm.

**Figure 2:**
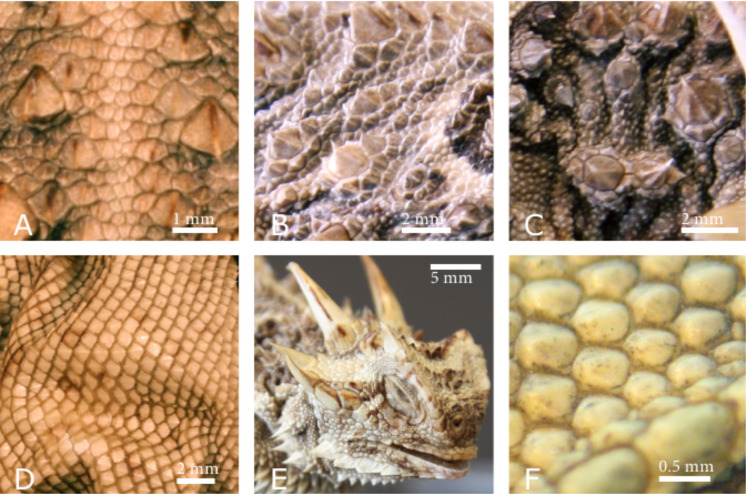
Different sizes and morphologies of scales of *Phrynosoma cornutum*. (A) Dorsal (back) scales along the medial line. (B) The lateral back is characterised by thorn like scales surrounded by small polygonal scales. (C) In the lateral neck region the thorns are mainly circular and are surrounded by small circular scales. (D) Ventral scales are diamond shaped and rather regular. (E) The scales on the head exhibit an enormous variety with respect to the morphology and size. (F) The scales on the chin are regular and almost circular.

The macroscopic body shape differs remarkably (Figure 1). While *Phrynocephalus arabicus* is an elongated smooth lizard, the bodies of *Phrynosoma cornutum* and *Moloch horridus* are covered with thorns or spikes, respectively, on their dorsal and lateral surfaces. The only common feature is a rather broad body [1]. The macroscopic morphology of the scales differs dramatically in the three species but also for one species with respect to the exact location on the body. This is shown for *Phrynosoma cornutum* in Figure 2. Clearly the scales can be regular or irregular, polygonal or almost circular. The average scale area ranges from below 0.02 mm^2^ to above 2.5 mm^2^. Some scales form a spine like caudal (tailwards) end whereas others have a smooth caudal edge. Also for the other two species, the size and shape of the scales differ in dependence on the body location (data not shown).

**Figure 3:**
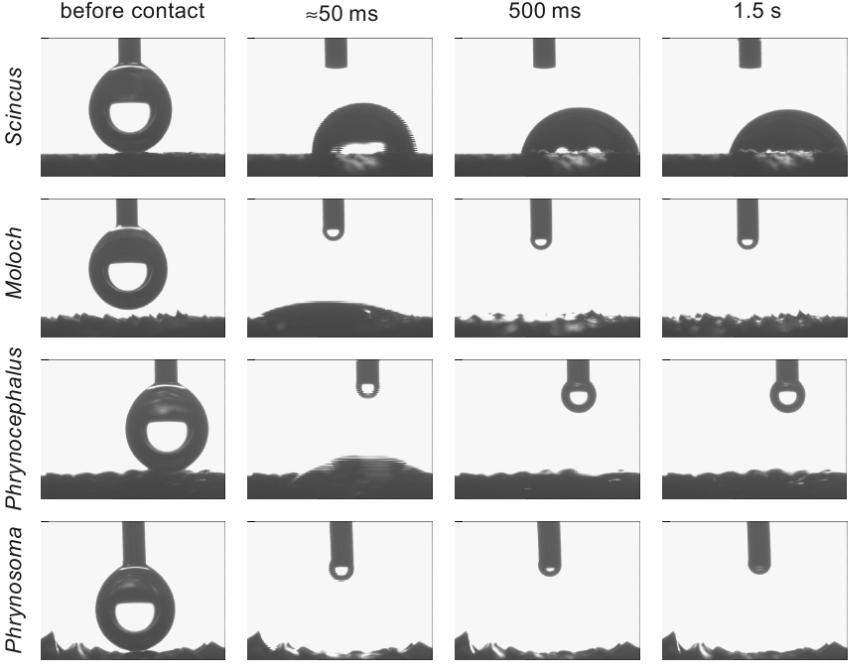
Water spreading on the reptiles' surfaces. A droplet of 5 µl was applied through a syringe and brought into contact with the surface by the use of a micro manipulator. While on the non-moisture harvesting lizard *Scincus scincus* the droplet hardly changes over time, an almost immediate spreading of the droplet on the moisture harvesting animals, i.e., *Moloch horridus*, *Phrynocephalus arabicus* and *Phrynosoma cornutum* can be seen. The images were recorded with a frame rate of 18 frames per second. Even in the first frame after contact, thus at about 50 ms after the droplet contacts the surface, the droplet is mostly spread and within 2 s the water is completely spread on the surface structure.

The common feature of the scales, independent of form or species, is the wettability. Application of a water droplet onto the integument leads in all three species to an almost immediate spreading of the water as shown in Figure 3A–C and in the supplementary videos (Supporting Information Files 1–3). In contrast to the integument of lizards that do perform moisture harvesting, the droplet hardly spreads as shown in Figure 3D for a specimen of the so called sandfish (*Scincus scincus)*.

Although the velocity of the water spreading varies slightly throughout the body, we found absolutely no correlation of the wetting behaviour and any macroscopic geometric parameter of the scales in the three investigated species (data not shown). Thus, either material properties or the micro ornamentation of the scales induce the high wettability.

### Contact angle and microscopic morphology

**Figure 4:**
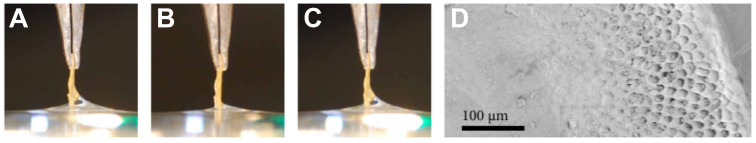
Scale of *Phrynosoma cornutum* dipped into deionised water. The freshly prepared scale (A) exhibits asymmetric behaviour. While on the inner side (left) a small meniscus, i.e., a large apparent contact angle of about 60° is formed, the outside of the scale (right) is wetted with an apparent contact angle of below 10°. If the experiment is repeated with a throughout dried scale (B), the difference of the inside and outside is less pronounced. After immersion of the scale in the water the scale again behaves like a freshly prepared scale (C). While the inside of this particular scale is completely flat (not shown), the outer surface exhibits a distinct micro ornamentation (D). Towards the edge (right) of the scale a honeycomb like structure is visible, while the centre of the scale is hardly structured.

To quantify the wetting properties we attempted to measure the apparent contact angle. Measuring the apparent contact angle of the non-moisture harvesting lizards such as *S. scincus* was simply performed by using a commercially available contact angle meter and gave an apparent contact angle of 76 ± 5° (*n* = 7) on the dorsal and 59 ± 7° (*n* = 7) on the ventral scales, respectively. Other different non-moisture harvesting lizards were measured and yielded similar results (data not shown). However, the measurements on the moisture harvesting species were more difficult since water spreads out almost immediately. Thus, the measurements were performed by dipping a large scale vertically into water. For this purpose, a scale, i.e., the keratinised Oberhäutchen was removed from a dead *Phrynosoma cornutum* from the so called beard-region. There we found the largest scales of about 2.5 mm^2^. This scale was dipped into water and we found asymmetric behaviour of the liquid (Figure 4A). While on the inner side of the scale a small meniscus was formed suggesting an apparent contact angle of about 60° to 70°, as measured by hand from the photo, the meniscus on the outer side is much higher forming an apparent contact angle of below 10°, rendering this surface super hydrophilic.

**Figure 5:**
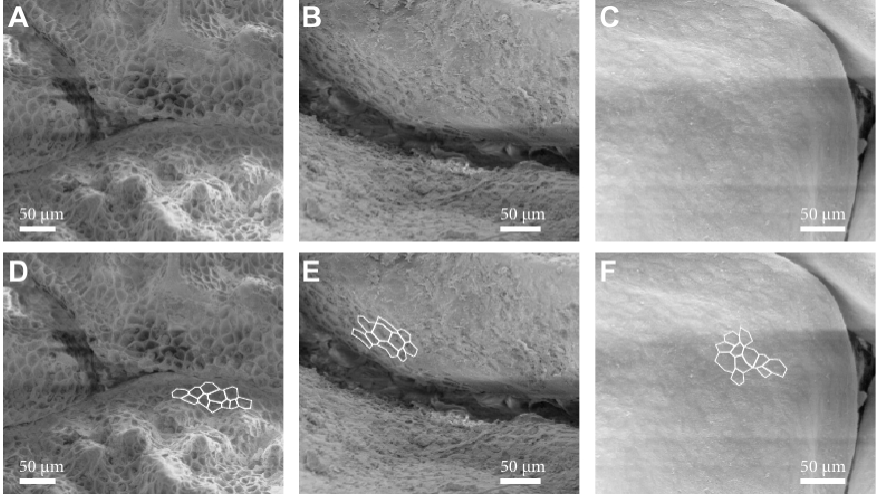
Micro ornamentation of the scales of the three investigated lizard species. (A) *Moloch horridus* shows the honeycomb like micro ornamentation virtually all over all scales. (B) *Phrynosoma cornutum* shows clear honeycomb like micro structures, but typically mainly at the periphery of the scales. (C) *Phrynocephalus arabicus* the only honeycomb like structures appear like dimples. (D) (E) and (F) show the images of A–C with some of the micro ornamentations marked for better orientation.

Interestingly, if the scale was completely dried in a desiccator containing silica gel before the experiment, the wetting was much less pronounced (Figure 4B). However, if the scale was completely immersed once and the experiment repeated, the same results as with the freshly removed scale could be obtained repeatedly (Figure 4C). When examined in the scanning electron microscope (SEM), the scale used for the above described experiment clearly revealed micro ornamentation on the outer side as previously described in the literature [14], whereas the inner side shows no micro ornamentation (Figure 4D). On the outer part, i.e., closer to the edge of the scale honeycomb like structures are visible on the outer surface. These structures are a more or less common feature of the scales of all three investigated species (Figure 5). Measurements with the SEM revealed that *Moloch horridus* exhibits these structures with a diameter of about 10 µm to 20 µm and a depth of about 5 µm in all scales (Figure 5A), while for *Phrynosoma cornutum* structures with a diameter of about 15 µm to 25 µm and depth of roughly 5 µm are mainly found at the scales' periphery (Figure 5B). *Phrynocephalus arabicus* has the least pronounced honeycomb structure. Here only dimples with a diameter of about 20 µm to 30 µm and a depth of about 1 µm are visible (Figure 5C). The depth was estimated from SEM-images with a viewing angle of less than 10° to the integument surface.

**Figure 6:**
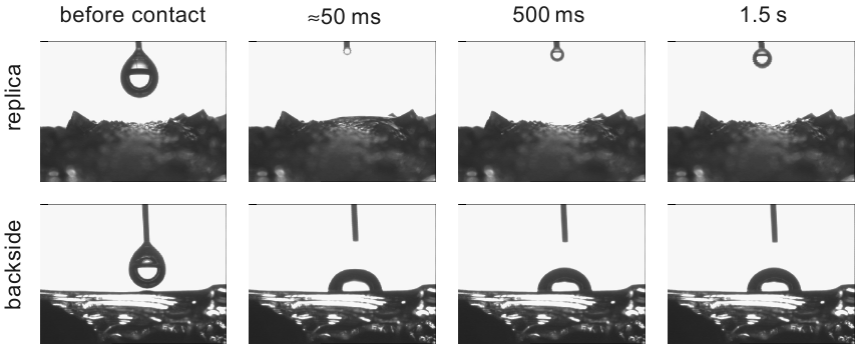
Water spreading on epoxy replica of moisture harvesting reptiles. A 5 µl droplet of water was applied to the epoxy replica of the medial back of the moisture harvesting species *Phrynosoma cornutum*. Similar to the situation on the native surface (see Figure 3), the water spreads at high velocity. The epoxy resin is not very wettable as can be seen when a 5 µl droplet is applied to the backside of the replica which exhibits an unstructured surface. The droplet stays perfectly constant forming a contact angle of about 80°.

To test further whether these structures are responsible for the reduced apparent contact angle, we manufactured epoxy resin replicas of the surfaces of different body parts of the investigated species. The unstructured epoxy resin has a contact angle of 79°, which is slightly higher than the scale material. According to electron microscopy, these replicas have a good resemblance to the surface structure (see Supporting Information File 1). As expected, the replicas did not exhibit an improved wetting behaviour if they were dried before the experiment. Under these conditions the contact angle is almost identical to the contact angle of the unstructured resin (data not shown). However, if a droplet is applied for some time (ranging from several seconds to about a minute) onto the replica, the water spreads spontaneously (Figure 6). After this, if the replica is not dried throughout, the replica stays highly wettable in contrast to the unstructured resin as exemplified in the supplementary videos (Supporting Information Files 5–6).

It is generally known that structuring, i.e., increased roughness of a hydrophilic material results in a decreased contact angle [16]. Assuming a Wenzel-model [16–17] for the wetting of the lizard scales however, does not fully predict the observed effect. Structures with typical sizes significantly below the dimension of the applied water droplet would result in an apparent contact angle cos φ* = *r* cos φ where φ is the contact angle of the unstructured material and *r* is the ratio of the actual surface and the apparent surface. In our case the unstructured material exhibits a contact angle of about 70° and the structured material is almost perfectly wettable, i.e., the contact angle is below 10°. This would require *r* > 3 which is approximately the case for *Moloch horridus* (*r* ≈ 3) but not for *Phrynosoma cornutum* (*r* ≈ 2.3) or *Phrynocephalus arabicus* (*r* ≈ 1.4) as estimated from the above mentioned dimensions of the honeycomb structures. So the reduction of the contact angle for these animals cannot simply be explained by increased roughness alone. Thus it is tempting to assume the Cassie-model for liquid impregnating [18] to be valid, i.e., that the honeycombs and dimples respectively, allow for the formation of a stable thin water film within a dimple so that even with a contact angle of about 60–70° as given by the scale's material, an outspread water-scale-contact is stable. The Cassie-formula for the apparent contact angle on a composite material is given by cos φ^Cassie^ = γ_1_ cos φ_1_ + γ_2_ cos φ_2_. Here the effective contact angle φ^Cassie^ is dependent on the area fraction of component one (γ_1_) and component two (γ_2_) and the contact angles of material one (φ_1_) and two (φ_2_). If the dimples are filled with water, i.e., φ_2_ = 0, the area fraction of water in the dimples is 73% (i.e., the fraction of pure scale material is 27%) and is sufficient to make the surface superhydrophilic and explain the described hysteresis of the contact angle.

### Water condensation on the scales

It is debated in the literature whether or not condensation of water directly on the lizards' bodies plays a major role for moisture harvesting [2,4–5]. Reptiles are cold blooded, i.e., they adapt their body temperature to the surrounding air with some delay which is related to the body mass. Thus after a cold night, the body might be cold enough to allow condensation of water from warm air in the morning. The temperature difference between day and night in deserts is normally remarkably high. Together with the typical air humidities, it was found that the temperature difference should be sufficient for significant condensation on a lizard [2]. However, experimental results show that the amount of condensed water is not sufficient to satisfy the daily water need of lizards [5,19]. It must be noted here that these experiments did not fully resemble the natural conditions.

**Figure 7:**
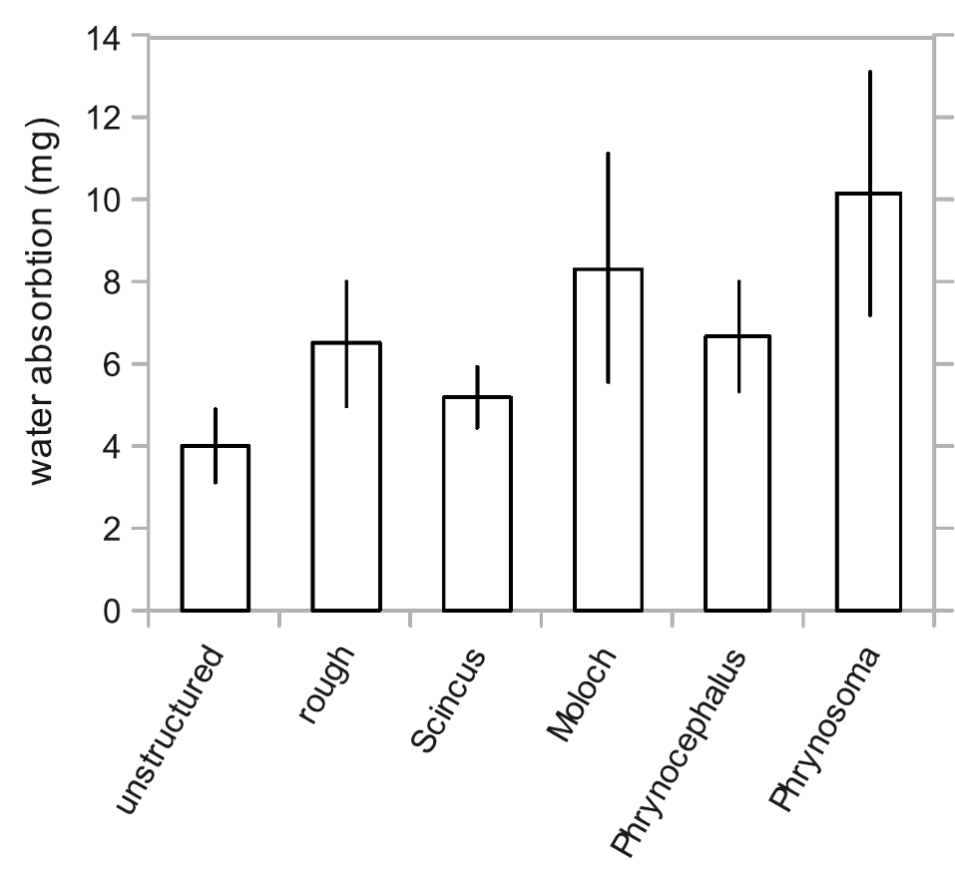
Water condensation on epoxy replicas of structured surfaces. The epoxy replicas were cut into disks with a diameter of 15 mm. These disks were thermally equilibrated to 20.5 °C and then held for 15 s in water-saturated air at a temperature of 80 °C. The amount of water was determined by the mass difference of each replica. Clearly structured surfaces allow for more condensation when compared with unstructured ones. Even an unspecific roughness introduced by polishing paper (roughness, *R*_a_ ≈ 3 µm) increased the amount of water, although not significantly (p = 0.070). The replica of a non-moisture harvesting lizard *Scincus scincus* exhibits no significant increase of the condensation (p = 0.093). The replicas of the moisture harvesting lizards *Moloch horridus* (p = 0.032), *Phrynocephalus arabicus* (p = 0.034) and especially *Phrynosoma cornutum* (p = 0.007) show a significantly increased water condensation. Significance was tested by use of a two sided t-test assuming different standard deviations for the samples.

To test the condensation behaviour, we measured the amount of condensed water on epoxy replica and on non-structured epoxy surfaces. The replicas were cut to a diameter of exactly 15 mm so all samples had about the same projected area for condensation. The replicas were thermally equilibrated at 20.5 °C. The mass was determined by weighing and then the replicas were put into 80 °C warm moisture saturated air for 15 s. After this time the mass was immediately determined again. The results are depicted in Figure 7. Clearly the total amount of water condensed on the epoxy resin is significantly higher on the replicas of the moisture harvesting lizards than on the unstructured resin as tested by an unpaired two-sided t-test. A replica of the non-moisture harvesting *S. scincus* hardly shows an improved condensation in comparison to the unstructured resin. If the resin is simply made rough (average roughness *R*_a_ ≈ 3 µm) by means of sand paper, the condensation is improved slightly, but not significantly. Thus the honeycomb structure increases the condensation by about 100% on average for the three lizard species under investigation. This might be caused by the increased roughness only, because a rough surface supports more condensation foci. However, if condensation had taken place leading to a water film in the honeycombs, the surface exhibits the above described high wettability which would then allow for further improved moisture harvesting from other water sources like rain, fog or dew.

The experiments on the removed scale as well as the condensation behaviour clearly indicate that a minimal moisture level on the scales is necessary in order to be effective. On the living animal condensation might further improve the initial wetting of the scales by the – in fact very small – water loss of the animal through the integument [5].

### Transport in the capillary network

**Figure 8:**
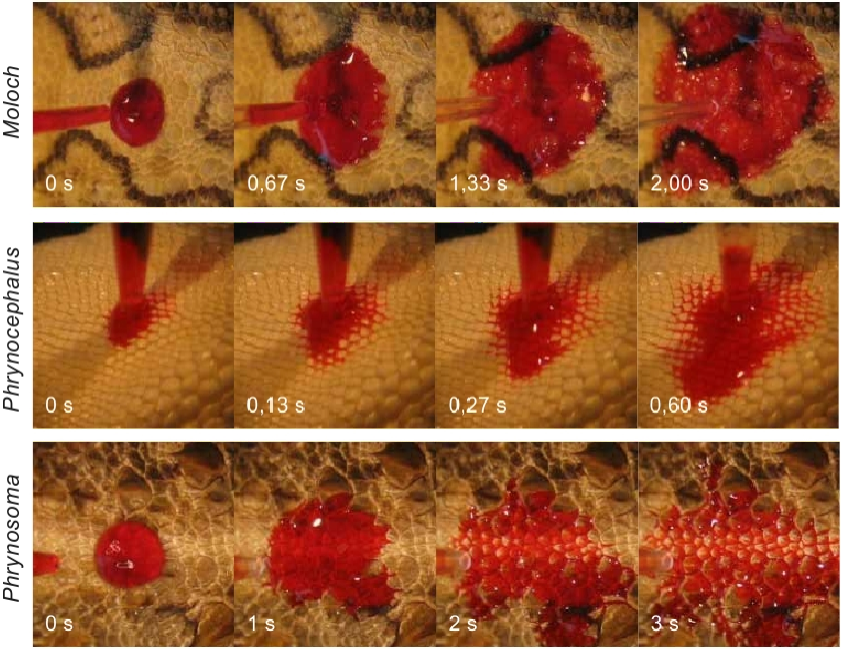
Water transport in interscalar capillaries. The behaviour of coloured water on the integument of the three investigated lizards is shown. The mouth is always located to the right. Note the colouring of the interscalar capillaries, best seen for *Phrynocephalus*. While the transport is about symmetrical for *Moloch* and *Phrynocephalus*, the velocity of the water transport is highest in direction to the mouth on *Phrynosoma*.

As stated earlier, initial wetting of the scales is necessary but not sufficient for successful moisture harvesting. The collected water has to be transported to the mouth of the animals as licking off the collected water from the integument is hardly possible due to the animals' anatomy. As shown in the image sequence in Figure 8 and in the supplementary videos (Supporting Information Files 7–9) water, when applied onto the animal, is adsorbed and immediately soaked into a capillary system which then transports the water passively.

Most interestingly while *Phrynocephalus arabicus* and *Moloch horridus* clearly show an almost symmetric water flow from the point of water application, i.e., similar transport velocities rostral (forward), caudal (backward) and lateral (sidewards), *Phrynosoma cornutum* behaves differently. As already clearly evident from the supplementary videos and as depicted in detail in Table 1, the water flows with significantly higher velocity rostral, i.e., to the mouth of the animal.

**Table 1:** Average water transport velocity^a^.

	average velocity (mm/s)		
	rostral	lateral	caudal

*P.a.*	1.94 ± 0.91	2.49 ± 1.01	2.57 ± 1.91
*P.c.*	3.15 ± 0.94	1.84 ± 0.24	1.61 ± 0.45
*M.h.*	2.18 ± 0.54	1.80 ± 0.43	2.15 ± 0.35

^a^The average transport velocity within the first 333 ms after the application of 7 µl of coloured water onto the different species, i.e., *Phrynocephalus arabicus* (*P.a.*), *Phrynosoma cornutum* (*P.c.*) and *Moloch horridus* (*M.h.*) is shown. The velocity was measured on different positions on the body with n ≥ 6 independent measurements for each condition. The values are rather constant within a species except for *Phrynosoma cornutum* (*P.c.*) where the velocity towards the mouth, i.e., rostral is significantly higher than for all other directions as determined by Wilcoxon-tests when applying a significance level of α = 0.05.

**Figure 9:**
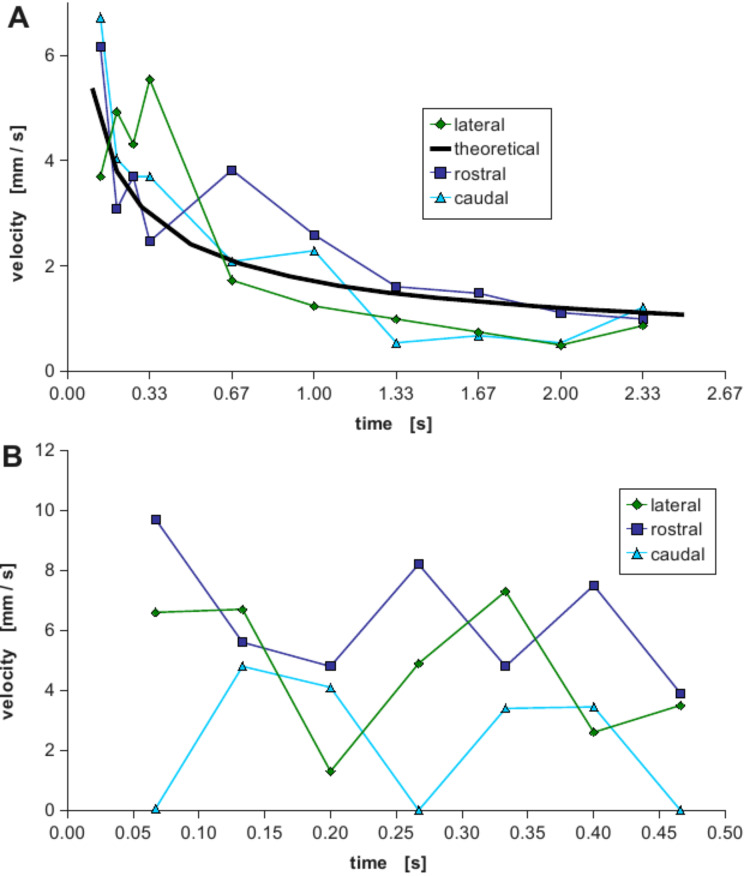
Water transport velocity in the capillary system. Two typical examples of results of a frame-to-frame analysis of videos showing the transport of coloured water in the capillaries are shown for *Phrynocephalus arabicus* (A) and *Phrynosoma cornutum* (B). The velocities were measured in direction to the mouth (rostral, dark blue squares), towards the tail (caudal, light blue triangles) and sidewards (lateral, green diamonds). For *Phrynocephalus arabicus* (A) no significant difference can be seen when comparing the velocities in the different directions. The theoretical behaviour described by the Washburn-equation (solid black line) fits the data quite well suggesting simple capillary transport uniformly flowing in all directions. In contrast for *Phrynosoma cornutum* the typical result is that the velocity has a highly oscillating behaviour and, most interestingly, the velocity towards the mouth is normally higher than tailwards or sideways.

Because of the highly irregular morphology of *Moloch horridus* a detailed analysis of the flow velocity over longer time intervals is time consuming. Thus we compared the flow velocity of *Phrynocephalus arabicus* and *Phrynosoma cornutum* in more detail. For this a frame-to-frame analysis of videos of water transport was performed yielding the velocity of the water in different directions over time. A typical result is depicted in Figure 9. For *Phrynocephalus arabicus* the velocity is almost identical in all directions over time (Figure 9A). The monotonous decrease can be modelled theoretically. Generally the water flow in a capillary system can be described by the Washburn-equation [20]

[1]
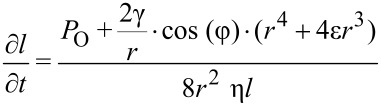


Here *l* is the distance the fluid penetrates into the capillary tube, *t* is time, *P*_O_ is the total outer pressure, i.e., the atmospheric pressure plus the hydrostatic pressure acting on the liquid. γ is the surface tension, φ is the contact angle of the liquid on the unstructured capillary material, *r* is the radius of the capillary, η is the viscosity of the liquid, and ε is the coefficient of slip.

The morphology of the capillaries was determined by microscopic analysis of semi-thin sections from all investigated species. We found that in between the scales semi-tubular channels are located (Supporting Information File 2) and we could measure the dimensions.

Because the total penetration length *l* of the liquid is hard to measure accurately in an irregular capillary system, we measured the liquid's velocity, i.e., ∂*l*/∂*t* in dependence on the time *t* by analysing videos on a frame-to-frame basis. For that the above ordinary differential equation was integrated numerically. By approximating the capillaries by tubes assuming the radius to be 30 µm and by introducing the parameters according to the literature and the above measurements (i.e., *P*_O_ = 0 as the species were almost horizontally, γ = 72.5 mN/m, φ = 60°, η = 1mPa·s) and by least square fitting the unknown coefficient of slip ε to be 0.1, one obtains the theoretical curve depicted as solid black line in Figure 9A. This curve resembles the measured values very well, thus normal capillary transport takes place in *Phrynocephalus arabicus*. The transport mechanism is homogeneous and the animal's mouth serves as a water sink. Thus when the capillary system is filled, water is ingested and thus sucked through the capillary system from all over the body, similar to the capillary system in plants where transport takes place via vessels due to evaporation of the water at the leaves which serve as a sink.

However, as depicted in Figure 9B the velocity behaviour for *Phrynosoma cornutum* differs dramatically and cannot be described by the above theory when assuming an uniform capillary system. In our microscopic analysis of semi-thin sections we found that indeed the dimensions of the capillaries' cross sections vary remarkably from 30 µm to 300 µm. Furthermore the wall structure shows some morphological details (dimples, protrusions, folds) which might be responsible for the directed flow [16] and which will have to be analysed in detail in future studies.

## Conclusion

Taken together we found that the convergently evolved honeycomb like micro structures on moisture harvesting lizards render the surface superhydrophilic. The contact angle of the unstructured material is 60–70° whereas the contact angle for the structured material is below 10°. This effect could be mimicked by polymer replicas of the lizards' surfaces clearly showing that the effect is not a material property but due to the structure. The structure can be supposed to hold a thin water film stable rendering the contact energy for further water to be decreased. The initial moisture necessary for this effect can be easily obtained by condensation since on the structured surfaces condensation is improved by about 100%. The collected water is effectively transported by an interscalar capillary network towards the mouth of the lizards. The mouth serves as water sink so that water will be soaked from the whole body's surface by capillary forces. In the case of *Phrynosoma cornutum*, the capillary effect is enhanced towards the mouth which terminates in a directed water transport.

## Experimental

Photographic images of the lizards were taken with a Canon EOS 350D (Canon Inc., Tokyo, Japan) with either the original telephoto lens or a 50 mm macro lens. The auto exposure setting was used without flashlight.

For SEM-imaging, tissue samples (approx. 1 × 3 mm) from different body regions of alcohol fixed museum specimen (Zoologisches Forschungsmuseum Alexander Koenig (ZFMK) in Bonn) of the lizards under investigation were taken. These samples were fixed overnight in 4% (v/v) glutardialdehyde in 70% ethanol followed by dehydration in an ascending alcohol series (90%, 60 min; 96%, 60 min; 99.8%, 60 min twice; 100%, 2 days). After washing three times for 20 min with hexamethyldisilazane the samples were dried at room temperature for 3 days. The samples were sputter-coated without further treatment with gold and observed using a Stereoscan S604 SEM (Cambridge Instruments, UK). Images were digitally recorded with an attached i-scan digitizer (ISS Group Services Ltd., Manchester, UK) with an image acquisition time of 50 s.

For determination of water behaviour on the animals' surfaces two different approaches were used. A commercially available contact angle meter (DSA-10, Krüss, Hamburg, Germany) was used applying droplets of about 5 µl of deionised water and observing the behaviour with an integrated video camera. Alternatively, droplets of 4–7 µl of deionised water containing the red colourant 0.5% (w/v) Ponceau S Red and the behaviour of the droplet observed by a Canon camera using the video mode. The image analysis was performed using the software GIMP (version 2.6.8) which allows for the automatic recognition of the red colour and for morphometric measurements.

For production of epoxy, replicas of a negative form was obtained from the animals using dental moulding paste VPS Hydro (Henry Schein Inc., Melvolle, USA). As alcohol fixated specimen from the ZFMK were used, the animals were initially dried for 30 min. A droplet of about 2 cm diameter was applied onto the integument and pressed onto the animal by use of a 10 mL petri dish. After hardening, the negative form was removed carefully from cranial to caudal. The negative form was filled with epoxy resin (Toolcraft, Conrad Electronic, Hirschau, Germany). The ratio of resin to hardener was 10:4. The resin was degassed in a desiccator for 5 min. The quality of the replicas was checked by SEM.

For condensation measurements, replicas were cut into discs of exactly 15 mm diameter. The samples were initially weighed and equilibrated at room temperature (20.5 °C) before holding them in a moisture saturated atmosphere at 80 °C. The weight increase was determined immediately.

For histological analysis of the integument, samples of approximately 1 × 3 mm size were fixed in 70% ethanol containing 2% (v/v) glutardialdehyde and 2% (v/v) formaldehyde. The samples were dehydrated in an ascending alcohol series (3 × 15 min 70%; 15 min, 80%; 15 min, 90%; 15 min 96% and 3 × 30 min, 100%). The samples were put into LR-White resin (London Resin Company Ltd., Berkshire, London) at 4 °C overnight. The resin was changed to new LR-White and allowed to polymerise at 60 °C for 48 h. The samples were cut into 7 µm thick slices using an OM U3 microtom (Reichert, Wien, Austria) stained with Methylene Blue and investigated with a standard optical microscope.

## Supporting Information

File 1 Application of a 5 µl droplet of deionised water onto the venter of *Moloch horridus*.

File 2 Application of a 5 µl droplet of deionised water onto the back of *Phrynocephalus arabicus*.

File 3 Application of a 5 µl droplet of deionised water onto the back of *Phrynosoma cornutum*.

File 4 SEM-image of *Moloch horridus* and the corresponding epoxy replica. Clearly the general morphology as well as the honeycomb-like micro ornamentation are well reproduced.

File 5 Application of 5 µl of deionised water onto a epoxy replica of the back of *Phrynosoma cornutum*.

File 6 Application of 5 µl deionised water onto the flat back side of the same replica as used in File 6.

File 7 Application of a 7 µl droplet of coloured deionised water onto the venter of *Moloch horridus*.

File 8 Application of a 7 µl droplet of coloured deionised water onto the back of *Phrynocephalus arabicus*.

File 9 Application of a 7 µl droplet of coloured deionised water onto the back of *Phrynosoma cornutum*.

File 10 Semi-thin histological sections through the integument of *Phrynosoma cornutum.* Black: spaces of the capillary system, due to overlapping scales. Different dimensions and wall morphologies can be observed.
