# Supplementary figures and images for: Moisture harvesting and water transport through specialized micro-structures on the integument of lizards
Source: Beilstein J Nanotechnol. 2011 Apr 13;2:204–14. doi: 10.3762/bjnano.2.24 (PMC3148043; doi:10.3762/bjnano.2.24)

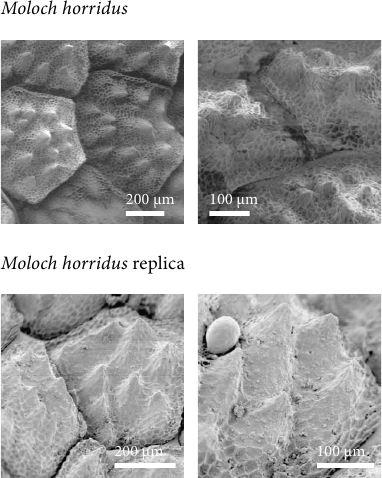

Supplement: File 4 — SEM-image of Moloch horridus and the corresponding epoxy replica. Clearly the general morphology as well as the honeycomb-like micro ornamentation are well reproduced. [file Beilstein_J_Nanotechnol-02-204-s004.tif]

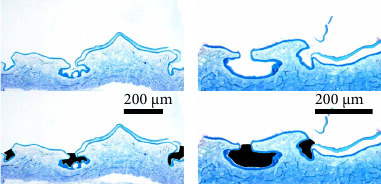

Supplement: File 10 — Semi-thin histological sections through the integument of Phrynosoma cornutum. Black: spaces of the capillary system, due to overlapping scales. Different dimensions and wall morphologies can be observed. [file Beilstein_J_Nanotechnol-02-204-s010.tif]
